# PrePhyloPro: phylogenetic profile-based prediction of whole proteome linkages

**DOI:** 10.7717/peerj.3712

**Published:** 2017-08-28

**Authors:** Yulong Niu, Chengcheng Liu, Shayan Moghimyfiroozabad, Yi Yang, Kambiz N. Alavian

**Affiliations:** 1 Department of Medicine, Division of Brain Sciences, Imperial College London, London, United Kingdom; 2 Key Lab of Bio-resources and Eco-environment of Ministry of Education, College of Life Sciences, Sichuan University, Chengdu, Sichuan, China; 3 School of Medicine, Department of Internal Medicine, Endocrinology, Yale University, New Haven, CT, United States of America; 4 Department of Periodontics, West China Hospital of Stomatology, Sichuan University, Chengdu, China; 5 Department of Biology, The Bahá’í Institute for Higher Education (BIHE), Tehran, Iran

**Keywords:** Linkage prediction, Whole proteome, Phylogenetic profile

## Abstract

Direct and indirect functional links between proteins as well as their interactions as part of larger protein complexes or common signaling pathways may be predicted by analyzing the correlation of their evolutionary patterns. Based on phylogenetic profiling, here we present a highly scalable and time-efficient computational framework for predicting linkages within the whole human proteome. We have validated this method through analysis of 3,697 human pathways and molecular complexes and a comparison of our results with the prediction outcomes of previously published co-occurrency model-based and normalization methods. Here we also introduce PrePhyloPro, a web-based software that uses our method for accurately predicting proteome-wide linkages. We present data on interactions of human mitochondrial proteins, verifying the performance of this software. PrePhyloPro is freely available at http://prephylopro.org/phyloprofile/.

## Introduction

The development of sequencing technologies has facilitated the access to whole genomic information from numerous organisms. Despite successful small-scale attempts to identify protein–protein interactions in a limited number of model organisms (Ewing et al., 2007; Li et al., 2004; Tarassov et al., 2008), determining genome-wide linkages remains a challenge. Phylogenetic profiling, by comparing genome sequences across different species, makes it possible to explore whole-proteome protein linkages (Pellegrini et al., 1999). This method is based on the assumption that functionally related proteins are likely to have evolved in a correlated manner. Several studies have successfully employed phylogenetic profiling to identify novel members of protein complexes (Avidor-Reiss et al., 2004; Dey et al., 2015; Gabaldon, Rainey & Huynen, 2005), expand known pathways (Li et al., 2014), and analyze non-coding elements (Tabach et al., 2013a).

Based on occurrence information across different species, two main groups of prediction algorithms have been proposed (Kensche et al., 2008). In the first group, the phylogenetic profiles of paired proteins are directly compared by a “co-occurrency” method such as Hamming distance (Cheng & Perocchi, 2015; Pellegrini et al., 1999), Pearson correlation coefficient (Glazko & Mushegian, 2004), Jaccard similarity (Brilli et al., 2008; Jaccard, 1912; Yamada, Kanehisa & Goto, 2006), Fisher’s exact test (Barker & Pagel, 2005), and mutual information (Huynen et al., 2000; Wu, Kasif & DeLisi, 2003). Other normalization methods, including singular value decomposition (SVD) (Franceschini et al., 2015; Psomopoulos, Mitkas & Ouzounis, 2013) and normalized phylogenetic profile (NPP) (Sadreyev et al., 2015; Tabach et al., 2013a; Tabach et al., 2013b) of phylogenetic profiles before calculating the co-occurrence, have been proposed to reduce the rate of false positive predictions. Although the co-occurrence-based methods do not correct for the effect of phylogenetic bias or the non-independence of the profile values, they are widely used for predicting functional linkages mainly due to being very time-efficient. The second group is comprised of “model-based” approaches such as collapsing of subtree (Von Mering et al., 2005), tree-kernel (Vert, 2002), maximum likelihood (Barker & Pagel, 2005), and parsimony methods (Barker, Meade & Pagel, 2007). To account for the statistical non-independence of the profile values, the model-based methods use the phylogenetic tree to correlate the evolutionary processes (Kensche et al., 2008). Recently modifications of these methods have used sophisticated statistical models to infer gene gain and loss across a wide range of eukaryotic organisms (Dey et al., 2015; Li et al., 2014). These methods are dependent on the reliability of phylogeny and require lengthy computational times. Many of these phylogenetic profiling algorithms are not user-friendly and have low computational efficiency resulting in high false positive rates.

As increasing numbers of sequenced genomes have become available, a number of phylogenetic profile databases and tools for visualization of *Eukarya* phylogenetic profiles have been developed (Cheng & Perocchi, 2015; Cromar et al., 2016; Ott et al., 2012; Sadreyev et al., 2015; Szklarczyk et al., 2015). After considering the computational efficiency and prediction power of the current methods and tools, here we propose a method and online tool, called PrePhyloPro (PPP), which combines multiple co-occurrency measures and utilizes top rank thresholds to determine potential linkages. To identify human whole-proteome functional linkages, we constructed a comprehensive phylogenetic profile using 972 different species. We evaluated PPP with positive and negative reference datasets based on known human pathways and protein complexes. In comparison to conventional phylogenetic profiling methods, this method presented overall improvement, i.e., higher sensitivity and enhanced specificity in the receiver operating characteristic (ROC) curves. Moreover, an analysis of biological features of the predicted protein links from 3,697 human pathways and complexes, resulted in 21.7% overall true positive rate when the top rank was set as 400. We also developed a web-based server based on PPP to acquire and visualize human whole proteome predicted linkages.

## Results

### Prediction of whole proteome functional linkages

To construct comprehensive phylogenetic profiles, we included a wide range of eukaryotic and prokaryotic organisms with at least one organism in every known *Class* or *Phylum*, resulting in 972 different species (Table S1). We then implemented a new phylogenetic profiling method, named PPP, by combining multiple co-occurrency measures. Apart from physically interacting protein pairs, the predicted linkages potentially represent related components (sensors, regulators, and regulons) of signalling pathways and subunits of protein complexes.

To assess the performance of PPP, we compared this technique with eight conventional phylogenetic profiling methods, which were divided into three categories. The first category, comprised of co-occurrency methods, including Jaccard similarity (“Jaccard”), Pearson correlation coefficient (“Cor”), mutual information (“MI”) and Hamming distance (“Hamming”), relied on the evolutionary similarity or distance (Glazko & Mushegian, 2004; Kensche et al., 2008). The second category represented the gain and loss relationships of two proteins with additional phylogeny; we used maximum likelihood (“Tree”) (Barker & Pagel, 2005), Dollo parsimony distance (“Dollo”) (Kensche et al., 2008) as representatives of this category. The third category combined co-occurrency methods with normalized phylogenetic profiles such as NPP (Sadreyev et al., 2015; Tabach et al., 2013a; Tabach et al., 2013b) and SVD (Franceschini et al., 2015; Psomopoulos, Mitkas & Ouzounis, 2013). Because of the evolutionary conservation of protein complexes, the correlations of subunits in the same complex have been widely used as validation datasets (Barker & Pagel, 2005; Kensche et al., 2008; Ta, Koskinen & Holm, 2011; Zhou et al., 2006). We retrieved subunit composition information for 1,604 human protein complexes from the “comprehensive resource of mammalian protein complexes (CORUM)” database (Ruepp et al., 2010) and generated multiple control datasets (Tables S2–S4).

### Performance of PPP in predicting known linkages

**Figure 1:**
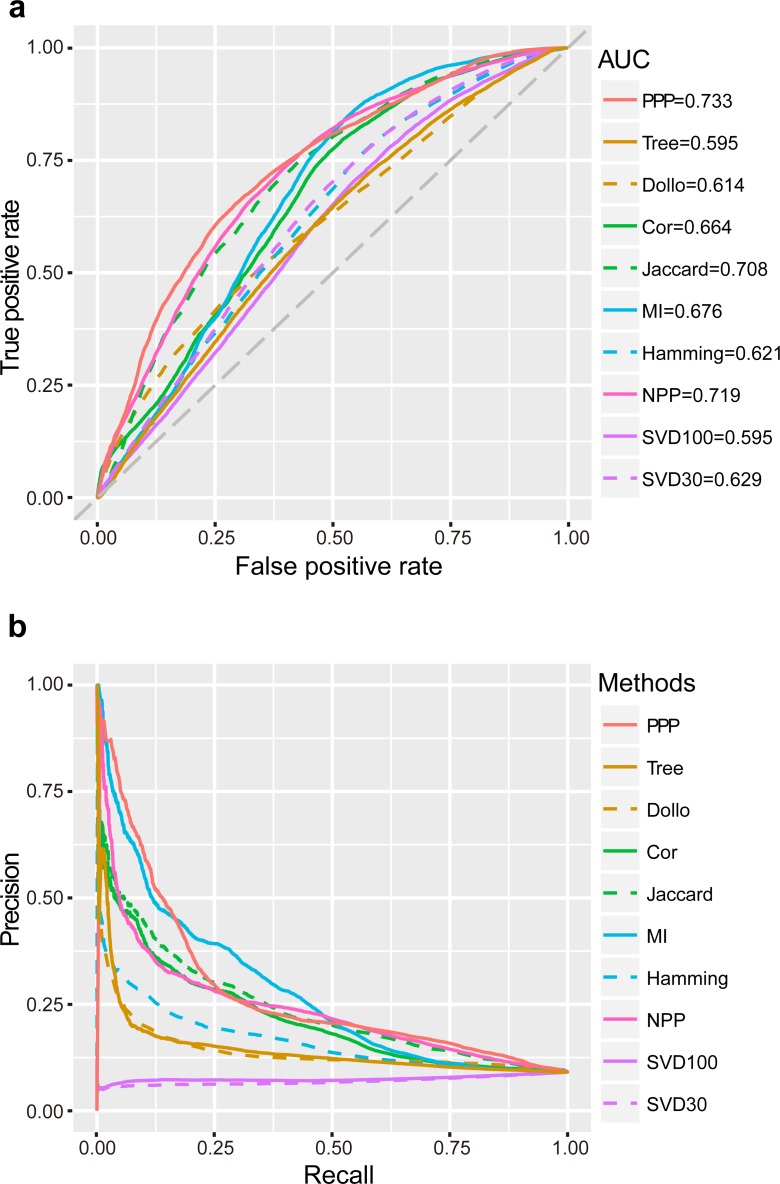
Performance of PPP. ROC curves (A) and PR curves (B) of PPP compared with Jaccard similarity (“Jaccard”), Pearson correlation coefficient (“Cor”), mutual information (“MI”), Hamming distance (“Hamming”), maximum likelihood (“Tree”), Dollo parsimony distance (“Dollo”), NPP normalization (“NPP”), and SVD normalization using all (“SVD100”) or top 30% (“SVD30”) of the unitary matrix, on a dataset comprising 57,114 positive linkages and 571,140 random protein pairs. The gray diagonal dash line is the random guess line.

ROC curves were plotted for the analysis methods after applying a series of relaxed thresholds. False positive rate (FPR) and true positive rate (TPR, also known as sensitivity) were calculated and represented in the *x*- and *y*-axis, respectively. A larger area under the curve (AUC) of ROC would indicate better reliability of the method. We observed that the AUC of PPP was the largest (0.73) in comparison to other conventional approaches. “Jaccard” had the third largest AUC (0.71), as this coefficient was one of the important tools used in PPP. In comparison to “Jaccard”, PPP had enhanced sensitivity upon increasing the FPR. For example, by changing the FPR to 0.20, the sensitivity of “Jaccard” was 0.46, whereas the sensitivity of PPP increased to 0.53, showing a noticeable improvement (Fig. 1A). “Cor” and “MI”, two similar correlation methods, had close AUCs (0.66 and 0.68, respectively). Interestingly, in comparison to PPP, “MI” displayed slightly higher sensitivity for the FPR values between 0.53 and 0.81. “Hamming” achieved a relatively low AUC of 0.62 (Fig. 1A). To determine the accuracy of positive predictions, i.e., potential functional linkages, we calculated the precision and recall (PR) for each method. In agreement with ROC curves, PPP showed a lower rate of decrease in the precision as the recall increased, indicating higher prediction of true positives comparing to the conventional methods. Similarly, more true positive predictions were detected by “MI” for the recall between 0.20 and 0.49 (Fig. 1B). Our results showed PPP identified overall more true linkages than each individual measure.

Recent studies have suggested that the model-based methods have higher discriminative power and better performance (Barker, Meade & Pagel, 2007; Barker & Pagel, 2005; Dey et al., 2015; Zhou et al., 2006). Other studies have questioned the superior performance of these methods mainly due to their reliance on the correctness of the annotation of genomes, which may not always be the case (Kensche et al., 2008). We, nevertheless, included the “Tree” (Barker & Pagel, 2005) and “Dollo” (Kensche et al., 2008) as representatives by using the likelihood ratio (LR) and the parsimony distance as measures, respectively. Our study found that the AUC and the decrease rate of precision were both lower in “Tree” and “Dollo” compared to PPP (Fig. 1). Our phylogenetic profiles included a wide range of both eukaryotic and prokaryotic species. The decreased precision rate of the model-based methods may be due to being arbitrarily applied to the phylogenetic profiles across a broad evolutionary scenario.

Sophisticated pre-processing and normalization methods are recently proposed to use sequence alignment bit scores instead of binary phylogenetic profiles, to accurately reflect the evolutionary relationships. For example, NPP (Sadreyev et al., 2015; Tabach et al., 2013a; Tabach et al., 2013b) and SVD (Franceschini et al., 2015; Psomopoulos, Mitkas & Ouzounis, 2013) combined the *z*-score and truncated unitary matrix with “Cor” and Euclidean distance, respectively. Our results showed that NPP achieved a significantly better performance than individual “Cor” correlation measures that had the second largest AUC (0.72). Similarly, setting the top percentage of unitary matrix as 30% (“SVD30”), would result in a higher AUC than that of the L_p_-norm based methods, including “Hamming” (Fig. 1).

To further examine the performance of PPP, we constructed another negative reference dataset with a different random seed in our program, confirming the reliability of the predicted linkages (Figs. S1A, S1B). To further validate the predicted linkages by PPP, we applied rebuilt positive linkages excluding large complexes (Figs. S1C, S1D) and an independent validation dataset described by Ta, Koskinen & Holm (2011) (Figs. S1E, S1F). The AUC of PPP was the largest in both cases, suggesting the robustness of this method. Furthermore, we validated predicted protein pairs that were present in a wide range of species by using the results from the MatrixMatchMaker (MMM) method (Bezginov et al., 2013; De Juan, Pazos & Valencia, 2013; Rodionov et al., 2011; Tillier & Charlebois, 2009) (Table S6). Among the MMM protein pairs, the ones with more homology showed a higher hit rate at stringent top ranks, indicating increased true linkage detection by PPP when both proteins of each pair are present in a wide range of species (Fig. S2).

**Figure 2:**
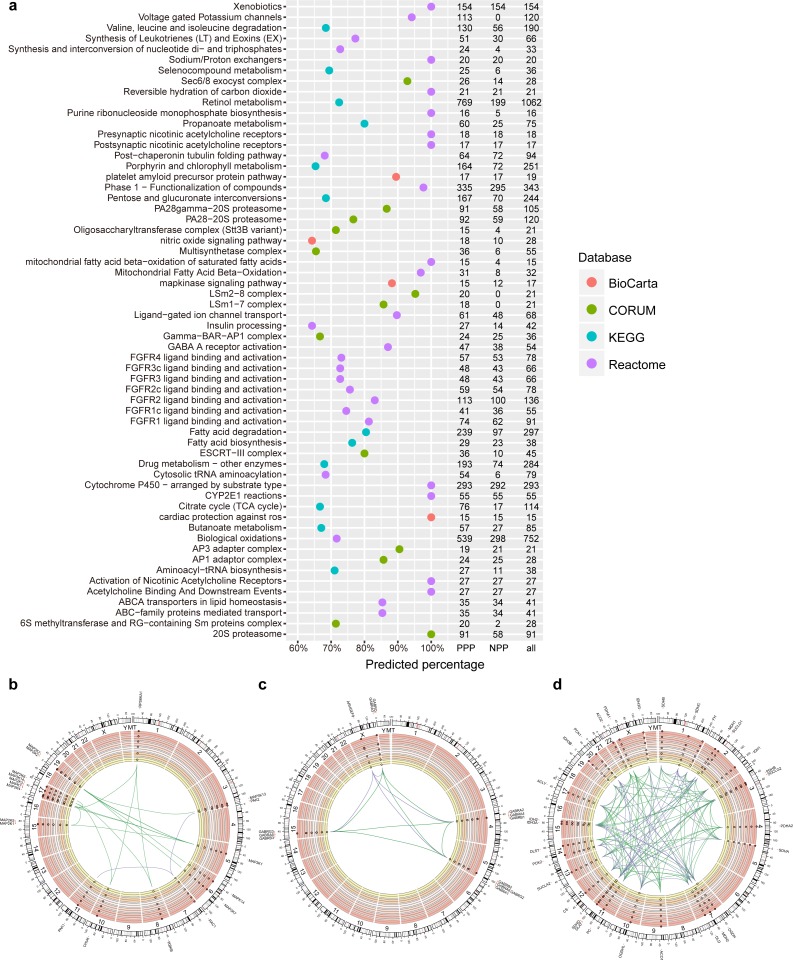
PPP predicted linkages in human pathways and complexes. (A) Top pathways and complexes with high predicted percentage (>50% and the number of predicted links is at least 15) in BioCarta, KEGG, Reactome, and CORUM databases. The number of PPP predicted (threshold 400, sensitivity 0.97), NPP predicted (threshold 0.73, sensitivity 0.97), and original linkages is presented on the right. (B–D) Selected Circos visualization of predicted linkages in the MAPK signaling pathway (B), GABA A receptor activation (C), and TCA citrate cycle (D). The outer ring shows the ideogram of human karyotype plus the mitochondria genome. The next six rings, coloured with yellow to dark red, show the present percentage for each protein. From outer to inner rings, points in each ring indicate the percentage in three Kingdom-size groups in *Eukaryota* (*Animals*, *Plants*, *Fungi*, and *Protists*), *Bacteria*, and *Archaea*. In percentage rings, the vertical axis ranges from 0 to 1, and the axis direction is from inner to outer space. The dash central line indicates the 50th percentile. In the centre, arcs indicate correlated relationships between paired proteins. The green arcs represent successfully predicted links according to the corresponding database. The purple arcs indicate false negative links. Outside the ideogram, proteins are annotated with symbols, and marked at corresponding genomic positions.

### Evaluating predicted linkages in human pathways and complexes

To evaluate the efficiency of PPP in predicting known linkages, we first generated a list of predicated linkages involved in human pathways or complexes based on five databases. We used Kyoto Encyclopedia of Genes and Genomes (KEGG), BioCarta, Reactome and NCI/Nature Pathway Interaction Database (NCI), for pathways analysis and CORUM to establish connections of proteins within complexes. The list included known linkages in 241, 247, 1,393 and 212 pathways in the KEGG, BioCarta, Reactome and NCI databases, respectively. We also included 1,604 different complexes through CORUM (Table S5). With the threshold of top interactions set as 400 (i.e., the top 400 protein pair phylogenetic profile correlations/similarities), PPP achieved an overall prediction rate of 21.7%. The method predicted more than 50% of the interactions in several pathways with at least 30 known linkages (Fig. 2A). The high ratios of predicted (PPP) and known/original (databases) links indicated the reliability of our approach. For example, the mitogen-activated protein kinases (MAPK) signalling pathway is comprised of a total of 17 known links, 15 of which were identified by PPP (Figs. 2A, 2B). We performed Circos visualization (Krzywinski et al., 2009), representing predicted links in three different pathways or complexes: MAPK signalling pathway (Fig. 2B), γ-aminobutyric acid (GABA) A receptor activation (Fig. 2C) and tricarboxylic acid (TCA) cycle (Fig. 2D).

PPP successfully identified the linkages within protein families, especially when members of the family share a common evolutionary pattern. It has been shown that the members of the MAPK signaling pathway arose at the dawn of eukaryotic evolution (Glatz et al., 2013) and may have orthologies in some bacterial species (Miller et al., 2010; Pereira, Goss & Dworkin, 2011). Our phylogenetic profiling confirmed the distribution of two members of this family, MAP2K and MAP3K, in almost all eukaryotes and some prokaryotes. This common evolutionary pattern was the basis for the detected linkages within the MAPK by PPP (Fig. 2B). Similarly, linkages between α subunits (GABRA1 to GABRA6), β subunits (GABRB1 to GABRB3), and γ subunits (GABRG2 and GABRG3) of the GABA A receptor were detected because of the similarity in their phylogenetic profile and their presence only in animals (Fig. 2C). By design, PPP uses co-occurrence as the main criterion for detecting linkages. The dissimilar phylogenetic distribution of two proteins, therefore, would translated into absence of interaction. This might result in the presence of false negatives, due to the involvement of evolutionary modules in pathways or complexes (Li et al., 2014; Pellegrini et al., 1999). For example, we failed to observe the linkages between ARHGEF9 and the other GABA A subunits, because homologs of α∕β∕γ subunits were exclusively present in *Metazoa* while ARHGEF9 homologs were also detected in *Fungi* (Fig. 2C). Modularity could be explained based on the selection for adaptation rate, where common evolutionary rates could force certain genes to evolve together and to maintain an interaction, preventing other genotypes (with similar phylogenetic profiles) from being included based on the difference in their rates of adaptation (Wagner, 1996).

Similarly, unlike the rest of the MAPK signalling pathway members, homologs of RAC1 were present mainly in eukaryotes, resulting in false negatives with respect to interaction with the other members of the MAPK pathway (Fig. 2B). Likewise, since PPP was based on calculating co-occurrences, it limited the correctly predicted linkages within the TCA cycle to the known evolutionary modules (Li et al., 2014) (for examples in “ACO1/ACO2/CS/DLST/SUCLA1/SUCLA2” and in “IDH3B/IDH3G/IDH3A”). Most of the true linkages to PCK1 and PCK2 were missed due to the same reason (Fig. 2D). Overall, our data suggests that PPP is suitable for predicting interactions between proteins that share common homologous distributions, but might be limited in detecting linkages between proteins that belong to different evolutionary modules in human pathways or complexes.

### Input of PrePhyloPro

We implemented PPP and comprehensive phylogenetic profiles into an intuitive and easy to use web-based software “PrePhyloPro” for whole proteome linkages prediction. PrePhyloPro could be used for detecting novel (physical) protein-protein interactions, for predicting new components of biological complexes, and suggesting potential new linkages in signaling pathways or metabolic processes.

PrePhyloPro is designed as a user-friendly tool that requires three steps. The first step is choosing algorithm parameters including the top rank threshold, BLAST *E*-value threshold, and the reference organisms. The top rank is the number of linkages (with the highest correlation coefficient values) corresponding to each queried protein. In the front page three options (0.001, 0.0005, and 0.0001) are provided for the BLAST *E*-value threshold. This threshold is applied to choose homologies among the 972 species in constructing phylogenetic profiles. Using a smaller top rank and BLAST *E*-value would result in less but more reliable linkages. Currently the PrePhyloPro software provides linkages prediction in two model organisms, *Homo sapiens* and *Arabidopsis thaliana*. Prediction in more species will be available, as future updates will be applied to the software. The second step is to set the size of protein names for the output plots as the default setting may not be suitable for studies with high or low number of queried proteins. In the last step, a table of query proteins in the “txt” or “csv” format will be uploaded onto the website for linkage prediction. The markup colours and aliases for query proteins could be added to this table to optimize visualization of output figures. Examples of input files are provided online.

### Output of PrePhyloPro

**Figure 3:**
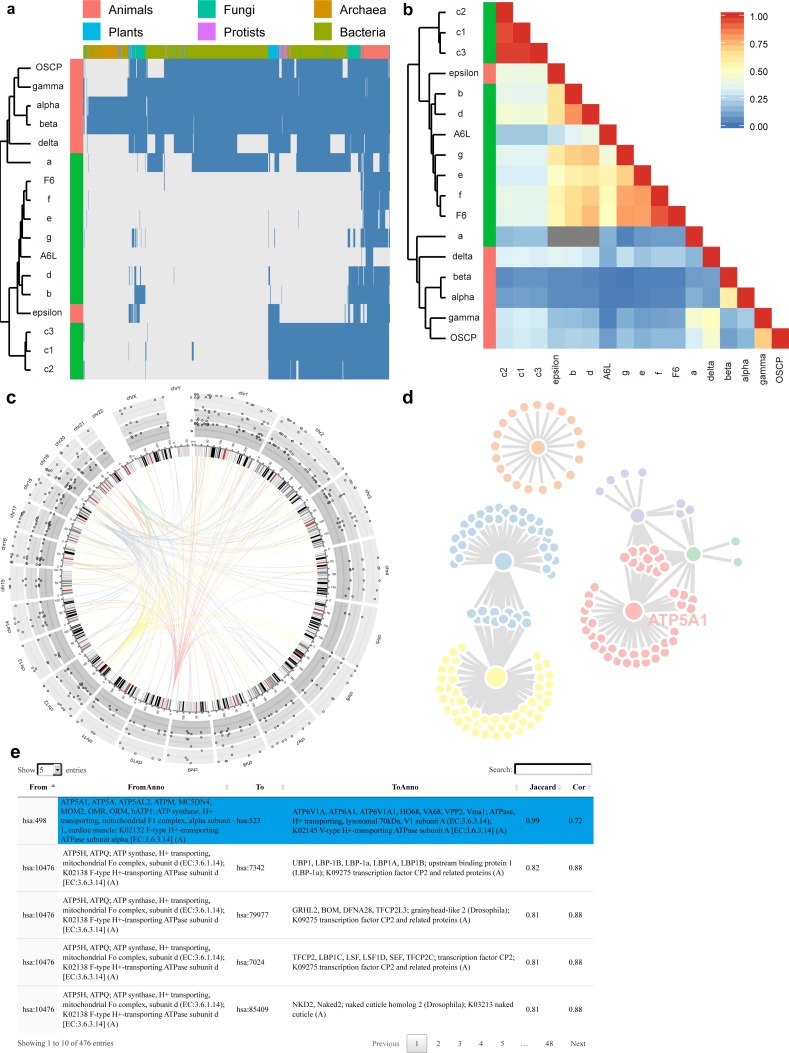
Outputs of PrePhyloPro using human F1Fo ATP synthase subunits as input proteins. (A–B) The phylogenetic profile plot (A) and the correlation matrix (B) of the F1Fo ATP synthase. The left colour bar indicates subunits of F1 (red) and Fo (green) regions. (C–D) The D3 interactive Circos plot (C) and the network (D) of predicted linkages of subunits in the F1 region. (E) The numeric table of predicted linages. The linkage between the α subunit and ATP6V1A is highlighted.

To minimize the processing time for each query, we have already calculated the co-occurrency for all protein pairs under the three BLAST *E*-value thresholds, and have saved them in backend databases. In total, PrePhyloPro takes less than 1 min to determine whole proteome search for linkages of 20 candidate (query) proteins. PrePhyloPro returns an integrated webpage including output figures and tables. As an example of an input protein set, we have used subunits of the human F1Fo ATP synthase (Fig. 3). The outputs for this set include the phylogenetic profile plot and correlation matrix of input proteins. In the phylogenetic profile plot, the top panel represents the 972 surveyed species, which are divided into six major taxa (*Animals*, *Plants*, *Protists*, *Fungi*, *Archaea*, and *Bacteria*). The left panel represents the input proteins (marked with user-defined colours) combined with a cluster dendrogram measured by Euclidean distances. The blue and gray bars in the phylogenetic profile plot correspond to presence or absence of homologies, respectively (Fig. 3A). The correlation matrix, as a complement to the phylogenetic profile plot, shows the Pearson correlation coefficient of paired input proteins that are colour-coded from blue (no correlation between any given profile pairs) to red (highly correlated profiles) (Fig. 3B). These two figures not only demonstrate homologous distributions of input proteins among an array of eukaryotic and prokaryotic organisms, but also indicate the evolutionary relationships within a query set. For example, the F1Fo ATP synthase is proposed to have evolved from at least two major parts, i.e., the catalytic core (F1) and the membrane-bound subunits (Fo) (Falk & Walker, 1988; Mulkidjanian et al., 2007; Rak, Gokova & Tzagoloff, 2011). As anticipated, PrePhyloPro clustered a majority of the subunits into two groups, corresponding to the F1 and Fo components (Figs. 3A, 3B).

The output of PrePhyloPro also includes the visualization of predicted linkages. We used the circosJS package (Girault, 2017) to create an interactive Circos plot, which integrates the chromosome location, homologous distribution, values of co-occurrence, and linkages. With the threshold of top rank and the BLASTP *E*-value set to 20 and 0.001, respectively, the predicted linkages of the F1 subunits of the ATP synthase (α, β, γ, δ, ε, and OSCP subunit) were displayed in Fig. 3C. An ideogram of normal karyotype plus the mitochondrial genome is plotted in the central ring. The outer three rings with grey background show the percentage of present homologies for each protein in *Bacteria*, *Archaea*, and *Eukaryota* (from the outer to the inner ring). Hovering over the points will show the corresponding present percentages. In the centre, connecting arcs represent the predicted links for the 6 subunits in F1 ATP synthase with user-defined colours (In Fig. 3C: α (blue), β (yellow), γ (red), δ (orange), ε (purple), and OSCP (green)). Hovering over the arcs will indicate the linkage partners, their chromosomal locations and Jaccard/Cor values.

To directly visualize network topology, PrePhyloPro generates an interactive linkage network using JavaScript D3 (Gandrud, Allaire & Kent, 2015). Nodes with more linked partners have a bigger size. Hovering over one node will enlarge its size and brings up its corresponding gene symbol. As an example, the α subunit (ATP5A1) of ATP synthase (Fig. 3D). In contrast to static visualization, the D3 network provides more interactive features, like dragging or pulling one node from a crowded group, which is particularly helpful for a large network with overlapping nodes.

The outputs of PrePhyloPro contain a table summarizing the predicted linkages. The “from” and “to” columns are composed of standard protein IDs of input proteins and the predicted interacting proteins, respectively. The proteins symbols and descriptions are also included in this table. The last two columns display the Jaccard similarity and Pearson correlation coefficient values sorted in a decreasing order. Hovering over one cell of the table highlights its row in a blue background. Other features include a search box at the right corner, the option for adjusting the number of entries (at the left corner), and the sort option (at the top of each column) (Fig. 3E). Output results and figures can be downloaded to local devices. The downloaded folder includes high quality figures. The correlation matrix and prediction linkages are stored in numeric tables that can be used for further analysis and validation.

PrePhyloPro identified the known linkages between the α∕β subunits and between the γ∕δ subunits of the F1 component of the synthase (Figs. 3C, 3E). The linkage between the α subunit and ATP6V1A (highlighted in Fig. 3E), a subunit of human *V*-type ATP synthase, was also identified by PrePhyloPro. These two proteins are believed to have evolved from the same ancestor by gene duplication (Iwabe et al., 1989; Shih & Matzke, 2013). Moreover, PrePhyloPro showed strong linkages (Jaccard similarity > 0.99) between the α∕β subunits and ATP-binding cassette transporters (ABC) family members (Table S7). Recent studies have shown functional linkages between the two sets of proteins. ABCB7 and nuclear genes of ATP synthase are both significantly down-regulated in SOD2 deficient erythroblasts under oxidative stress (Martin et al., 2011), while mutations of ABCD1 lead to the oxidation of α∕β subunits and defects in oxidative phosphorylation (Lopez-Erauskin et al., 2013). These studies suggest a possible regulatory relationship within ATP synthase and ABC family. Another interesting predicted partner of α∕β subunits was AFG3L2 (Jaccard similarity > 0.99). Mutations in the *Saccharomyces cerevisiae* homolog of AFG3L2, AFG3, inhibit the assembly of ATP synthase, suggesting a similar role of AFG3L2 in human (Guzelin, Rep & Grivell, 1996; Paul & Tzagoloff, 1995). Moreover, PrePhyloPro predicted the high correlations (Jaccard similarity >0.95) between α∕β subunits and adenylate kinase isoforms (AKs) (Table S7). Consistently, AKs maintain the cellular energy balance and collaborate in ATP synthesis, especially through coupling of the mitochondrial resident AK2 with the OXPHOS activity (Klepinin et al., 2016). Additionally, the transcription of AK2 and α∕β subunit are both enhanced by triiodothyronine (Severino et al., 2011). It has also been suggested that the transcription of α subunit and AK1 is regulated by PGC-1α, a master regulator of metabolism (Lucas et al., 2014). These studies confirm the potential of PrePhyloPro in predicting linkages based on co-evolution of proteins.

**Figure 4:**
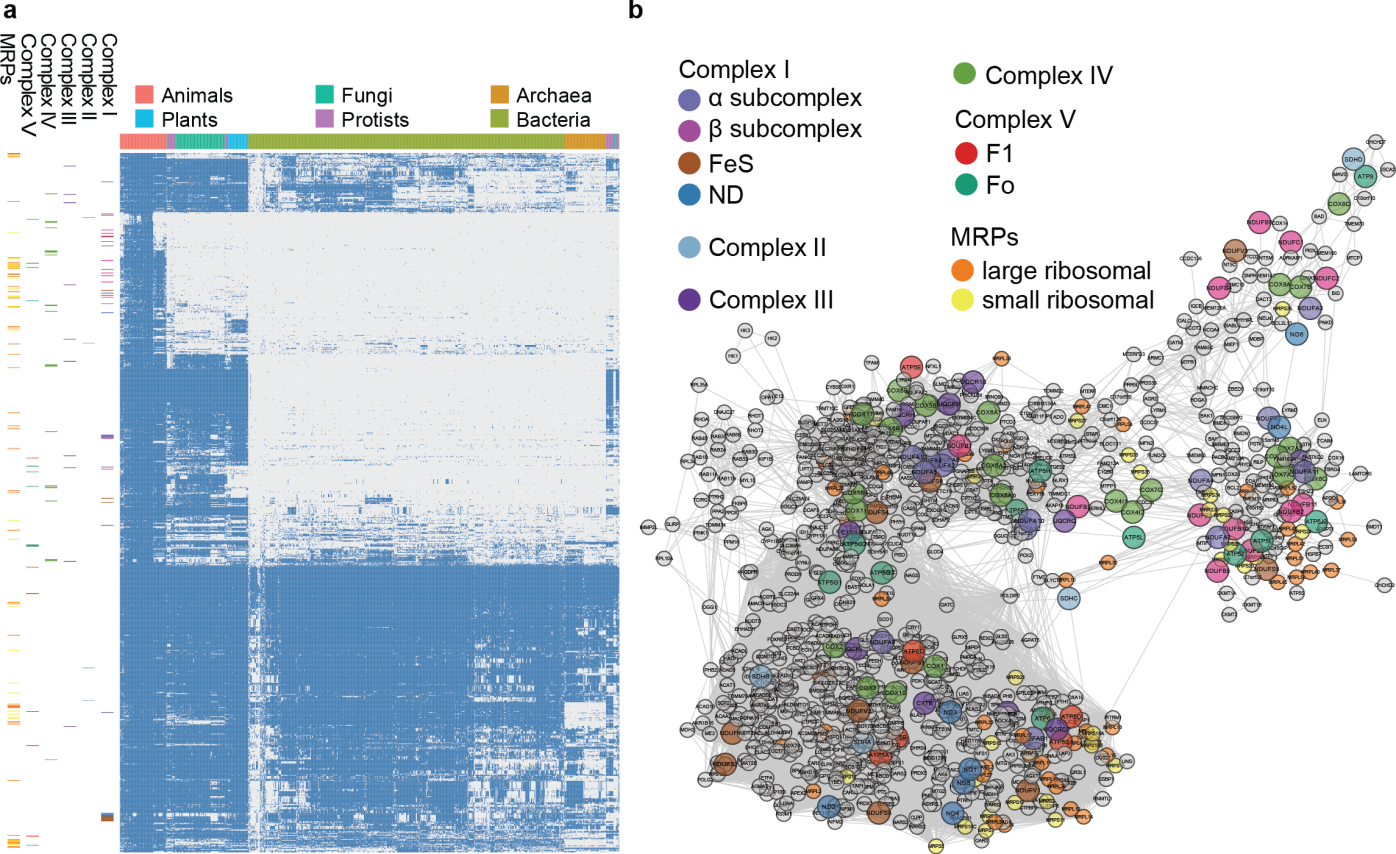
Phylogenetic profile and network visualization of human mitochondrial proteome. (A) Phylogenetic profile plot for 1,006 human mitochondrial proteins across 972 fully sequenced organisms. Blue and grey squares indicate the gene gain and loss, respectively. Hierarchical cluster is applied to both the organisms (columns) and proteins (rows). The organisms are organised into 6 taxa: *Animals*, *Plants*, *Fungi*, *Protista*, *Bacteria*, and *Archaea*. On the left, each small band indicates the corresponding subunits in complex I to V, and MRPs. (B) Predicted linkage network for human mitochondria proteins.

### Inferring evolutionary relationships from phylogenetic profiles of mitochondrial proteins

To evaluate the large-scale prediction power of PrePhyloPro, we used the entire human mitochondrial proteome containing 1,006 mitochondrial proteins (Pagliarini et al., 2008) as the input set. PrePhyloPro returned the list of predicted linkages and the phylogenetic profile plot of mitochondrial proteins (Fig. 4A). Only predicted linkages between a pair of mitochondrial proteins were selected for the purposes of visualization (Fig. 4B). Interestingly, after mapping the oxidative phosphorylation complexes (complex I to complex V) and mitochondrial ribosomal proteins (MRPs), we noticed that the subunits within a complex are dispersedly distributed in the phylogenetic profile figure (Figs. 4A, 4B). In agreement with previous studies (Li et al., 2014; Pagliarini et al., 2008), this indicated that mitochondrial proteins have originated from multiple modules during evolution. PrePhyoPro detected closer linkages among members of known evolutionary modules (Li et al., 2014) of mitochondrial complexes. For example, COX1, COX2, ATP6, ND1, ND3, and SDHA that are widely present among eukaryotic and prokaryotic species are gathered tightly in a subnetwork (Fig. S3). Confirming this result, a recent study showed that dietary lipid affects the expression of COX1, COX2, ATP6, and ND1 by common transcription factors such as the peroxisome proliferator-activated receptor (Eya et al., 2015). On the other hand, the group consisting of COX7A1, NDUFB1, ND6, and NDUFA1 was exclusively present in the *Metazoa* and was concentrated in a different subnetwork (Fig. 4B and Fig. S3). These results suggest that, in addition to detecting protein linkages, PrePhyloPro provides insight into the evolutionary relationships between paired proteins. Deciphering the evolutionary relationships within a query set would be useful for further exploring biological functions in pathways and complexes.

## Discussion

In this study, we implemented a phylogenetic profiling method named PPP to predict whole proteome linkages. PPP combined multiple co-occurrences and used top ranks to select most likely linkages. This method excluded solo proteins that have no connections with other proteins in prediction results, even when a stringent threshold was set to achieve more reliable linkages. Moreover, PPP displayed robustness in comparison to other conventional approaches. We are aware that factors, such as control datasets or the species chosen to construct phylogenetic profiles and trees, may contribute to the poor performance of some methods. For example, in comparison to PPP, “MI” displayed higher sensitivity in the ranges corresponding to large FPR, which may be due to the discrimination power of mutual information. But because “MI” is limited in making a distinction between the anti-correlating and correlating protein pairs (Steuer et al., 2002), positive predictions established with a high “MI” threshold might include negatively correlated pairs.

In our test datasets, model-based methods exhibited lower predictive power than co- occurrence methods. A major limitation of model-based methods is the underestimation of paired proteins that are both present in a wide range of species, resulting in lack of true positive predictions. For example, the α and β subunits of the F1Fo ATP synthase are two co-occurring proteins, as they are present in almost all living species (Gogarten et al., 1989). They physically interact with each other to form an α_3_β_3_ hexamer (Rubinstein, Walker & Henderson, 2003). “Tree” and “Dollo”, however, yielded an extremely low value indicating no linkages. The “Tree” method showed a rapid increase in precision to the maximum and remained constant at more stringent LR thresholds, suggesting higher prediction power of “Tree” with carefully chosen LRs (Barker & Pagel, 2005). Dollo parsimony distance is limited in eukaryotes, where horizontal gene transfers are rare events (Barker, Meade & Pagel, 2007; Kensche et al., 2008). Thus, model-based methods would have a better performance in predicting functional linkages specific to a *Class* or a *Phylum* rather than linkages that are conserved across a wide range of species.

Although PPP showed improvements in ROC curves, two kinds of inaccuracies still existed, which could be corrected by taking additional steps. The false negatives occurred when actually linked partners had different evolutionary rates. For example evolutionary constrains are not common in signalling and transcriptional pathways (Dey et al., 2015). Other than protein co-evolution based approaches, methods like weighted gene co-expression network analysis (WGCNA) is appropriate to detect proteins that share similar co-expression patterns (Langfelder & Horvath, 2008; Liu et al., 2015). On the other hand, in addition to setting stringent thresholds for the top rank, using network algorithms to filter PPP outputs might help in reducing the rate of false positives. This approach could include selecting hubs connecting to more input proteins by using centrality measurements (degree and betweenness).

Several online tools exist for phylogenetic profiling, for example STRING (version 10) using SVD (Szklarczyk et al., 2015), PhyloGene using NPP (Sadreyev et al., 2015), and ProtPhylo using “Hamming” (Cheng & Perocchi, 2015). PrePhyloPro based on PPP is a complementary online tool for whole proteome linkage predictions. PrePhyloPro includes several visualization methods, including the interactive Circos plot integrated metadata of the homology distribution, genome locations, occurrency values and prediction linkages.

## Methods

### Phylogenetic profiling

Protein sequences and annotation information from 972 different species were retrieved from the KEGG database (Kanehisa et al., 2006), including 276 eukaryotic, 614 bacterial, and 82 archaea organisms, as well as mitochondrial and chloroplast proteins. BLASTP (Camacho et al., 2009) was used to comparing 20,127 human proteins sequences with selected species. To construct the homology matrix, we used BLASTP *E*-value as the criteria, in which 1 denoted that homologies of human proteins found in the corresponding species, otherwise 0.

Four independent co-occurrence methods were used to evaluate the correlated relationship between a pair of proteins (Glazko & Mushegian, 2004; Kensche et al., 2008). For each pair of proteins across *n* species, for example $X,Y\in { \left\{ 0,1 \right\} }^{n}$, the Jaccard similarity is defined from co-occurrence of presences:

(1) \begin{eqnarray*}J \left( X,Y \right) = \frac{ \left\vert \left\{ i{|}{x}_{i}=1\cap {y}_{i}=1 \right\} \right\vert }{ \left\vert \left\{ i{|}{x}_{i}=1\cup {y}_{i}=1 \right\} \right\vert } .\end{eqnarray*}

The Pearson correlation coefficient is:

(2) \begin{eqnarray*}cor \left( X,Y \right) = \frac{\sum _{i=1}^{n} \left( {x}_{i}-\overline{X} \right) \left( {y}_{i}-\overline{Y} \right) }{ \left( n-1 \right) {S}_{X}{S}_{Y}} \end{eqnarray*}

where $\overline{X}$ is the sample mean of *X*, and *S*_*x*_ is sample standard deviations of *X*.

The mutual information is:

(3) \begin{eqnarray*}I \left( X,Y \right) =\sum _{x\in \left\{ 0,1 \right\} }\sum _{y\in \left\{ 0,1 \right\} }p \left( x,y \right) \log \nolimits \left( \frac{p \left( x,y \right) }{p \left( x \right) p \left( y \right) } \right) \end{eqnarray*}

where $p \left( x \right) $ is the probability of a symbol (0 or 1) appears in *X*.

The *L*_*p*_-norm is defined as:

(4) \begin{eqnarray*}{d}_{L}={ \left[ \sum _{i=1}^{N}{ \left\vert {x}_{i}-{y}_{i} \right\vert }^{p} \right] }^{1/p}\end{eqnarray*}

where *p* = 1 is the Hamming distance.

We applied two phylogenetic tree based method, maximum likelihood (Barker & Pagel, 2005) and Dollo parsimony distance (Kensche et al., 2008), as representations of model-based methods. In order to reduce the computational time, as well as achieving a balanced phylogenetic tree, we decreased two third of the bacterial species. The phylogenetic tree was constructed based on the small ribosomal RNA (16S/18S) sequences downloaded from the SILVA database (release 119) (Quast et al., 2013). After reducing the redundancy of ribosomal RNA sequences, a total of 522 species were selected including 243 in *Eukaryota*, 201 in *Bacteria*, and 78 in *Archaea*. The truncated ribosomal RNA sequences were aligned using the MAFFT program (Katoh & Standley, 2013), and the phylogenetic tree was generated by the RAxML program with default parameters (Stamatakis, 2014). LRs of the maximum likelihood method were calculated by the BayesTrait (Barker & Pagel, 2005). In the Dollo parsimony method, the gain/loss state is firstly reconstructed for each node of the phylogenetic tree, and then the Dollo parsimony distance is calculated as:

(5) \begin{eqnarray*}{d}_{\mathrm{Dollo}} \left( X,Y \right) =\sum _{i\in \mathrm{branches}} \left\vert \left( anc \left( {x}_{i} \right) -desc \left( {x}_{i} \right) \right) - \left( anc \left( {y}_{i} \right) -desc \left( {y}_{i} \right) \right) \right\vert \end{eqnarray*}

where $anc \left( {x}_{i} \right) $ and $desc \left( {x}_{i} \right) $ are the ancestral and descendant’s state of a branch (Kensche et al., 2008).

The NPP was used to normalize phylogenetic profiles, in which processed BLASTP bit scores were included (Sadreyev et al., 2015; Tabach et al., 2013a; Tabach et al., 2013b). In a bit score profile *P* with *n* proteins across *m* species, for each bit score, it was set as 1 if lower than the 70. Then for each protein *n*_*i*_, if its number of homologous across *m* organisms was lower than a threshold, e.g., 12, the protein is removed because of its poor conservation. Next the bit score *p*_*i*__*j*_ was normalized as $\mathrm{log}~2 \left( {p}_{ij}/{p}_{\max i} \right) $, where *p*_max*i*_ was the maximum bit score in the i-th row. The last step was to normalize bit scores across species. Specifically, the bit score *p*_*i*__*j*_ was normalized as $ \left( {p}_{ij}-{\mu }_{j} \right) /{\sigma }_{j}$, which was also known as the *z*-score, where *μ*_*j*_ and *σ*_*j*_ were the mean and the standard deviation of the j-th column, respectively. Compared to the original profile *P*, the NPP normalized profile ${P}^{{^{\prime}}} \left( {n}^{{^{\prime}}}\times m \right) $ had the same organism number *m*, but may contain less proteins.

Another normalization method was called SVD (Franceschini et al., 2015; Psomopoulos, Mitkas & Ouzounis, 2013). In a bit score profile *P* with *n* proteins across *m* species, for each bit score, it was set as 0 if lower than the 60. Then the bit score *p*_*i*__*j*_ was normalized as *p*_*ij*_∕*p*_max*i*_, where *p*_max*i*_ was the maximum bit score in the i-th row. The next step was to SVD of the profile following *P* = *U*∑*V*′, where *U* was the unitary matrix. The profile *P*′ was defined as the top trimming columns of *U*. Because the SVD predictions are sensitive to the “trimming” parameter (top percentages of the unitary matrix), we set this parameter as 100% and 30%. Similar to the second step of NPP, poor conserved proteins were marked in the original profile and removed in ${P}^{{^{\prime}}} \left( {n}^{{^{\prime}}}\times {m}^{{^{\prime}}} \right) $. The last step was Euclidean normalization of species in *P*′ as ${p}_{ij}/\sqrt{{\mathop{\sum }\nolimits }_{i=1}^{{m}^{{^{\prime}}}}{p}_{ij}^{2}}$. The SVD normalized profile *P*′ may have less organisms and proteins than those in the original profile *P*.

The Pearson correlation coefficient and Euclidean distance (*p* = 2 in Eq. 4) were applied to measure co-occurrence of paired proteins as described in NPP and SVD, respectively.

### PPP method

We combined co-occurrences to implement a new method named PPP to improve the prediction efficiency. PPP was inspired by “solo proteins” that did not link to any other proteins, when we conducted whole proteome linkages prediction. However, solo proteins may be not in existence, considering the huge size of protein interactions representing complex biological activities (Stumpf et al., 2008). One solo protein occurred when correlated relationships of this protein with others were all lower than a pre-defined threshold in co-occurrence methods. Especially, a stringent threshold set to get higher reliable linkages always yielded more solo proteins. Thus, instead of setting an arbitrary threshold of co-occurrency, PPP chose top-ranking *T* linkages for each protein.

We illustrated PPP by predicting whole proteome linkages among *n* proteins from a phylogenetic profile across *m* species. The first step was to roughly exclude linkages with negative correlations. For a given protein *n*_*i*_, the Pearson correlation with the other proteins was calculated and denoted as $cor \left( l \right) \in {V}_{cor}$. Similarly, the Jaccard similarity vector *V*_*J*_ was generated. We excluded proteins with negative Pearson correlations, because a negative number would imply the presence of one protein and the absence of another in a pair, which could not be considered as a functional link under an evolutionary scenario. In the next step, we recorded the rank in decreasing order of each element in the vector *V*_*J*_ and denoted as $rank \left( l \right) \in {V}_{J-\text{rank}}$. Finally, top T proteins were considered to be functionally linked to the proteins

(6) \begin{eqnarray*}{L}_{i}= \left\{ l{|}cor \left( l \right) \gt 0\wedge rank \left( l \right) \lt T \right\} \end{eqnarray*}

where *T* ranges from 1 to *n* − 1. Thus, for a given protein, it has at least one partner owning the largest co-occurrency. A smaller *T* yielded less but more reliable linkages.

The whole proteome functional linkages were calculated for proteins as the same procedural $L= \left\{ {L}_{1},\ldots ,{L}_{\mathrm{n}} \right\} $. The predicted linkages were considered as a symmetric relationship, which means that we neglected the linkage direction between paired proteins.

### Control datasets

We retrieved 1,604 human complexes from the CORUM database (Ruepp et al., 2010). To generate the positive references, we chose paired proteins in same complexes. The control dataset consisted a total of 57,114 positive linkages (Table S2) and 571,140 negative linkages (Table S3), which were constructed by randomly selecting two proteins located in different complexes. Moreover, the negative linkages were not allowed from the complexes that resided in the same subcellular position. To avoid an arbitrary judgment, we could randomly choose multiple negative linkage lists (Table S4). Because protein pairs in large complexes contributed a large proportion in positive linkages and bias the results, we excluded complexes containing more than 40 subunits and generated another control dataset with 29,189 positive linkages and 291,890 negative linkages. Moreover, we used the control datasets from Ta, Koskinen & Holm (2011), which included 26,525 positive linkages and a same number of negative ones.

Similarities or distances in co-occurrence and normalization methods, LRs in the maximum likelihood method, distances in the Dollo parsimony method, and top rank in PPP were used to generate series of thresholds. Under each threshold, the number of TP true positives (TP) and true negative (TN) represented the positive and negative predicted linkages, respectively. In contrast, false positive (FP) and false negative (FN) were erroneously detected as positive and negative linkages under certain thresholds. In ROC curves, we represented the FPR and TPR in the *x*-axis and *y*-axis, respectively, as:

(7) \begin{eqnarray*}FPR=1-specificity=1- \frac{TN}{N} \end{eqnarray*}

(8) \begin{eqnarray*}TPR=sensitivity= \frac{TP}{P} .\end{eqnarray*}

*P* and *N* represent the total number of positive and negative reference links, respectively. In PR curves, we defined the precision and recall as:

(9) \begin{eqnarray*}Precision= \frac{TP}{TP+FP} \end{eqnarray*}

(10) \begin{eqnarray*}Recall= \frac{TP}{TP+FN} .\end{eqnarray*}

To validate the linked proteins that were present across a wide range of species, we compared the PPP predicted linkages with the results generated by the MMM method, which evolves the largest common submatrix between paired proteins (Bezginov et al., 2013; Rodionov et al., 2011; Tillier & Charlebois, 2009). With the MMM threshold set as 12, a total of 6,422 co-evolved protein pairs were retrieved (Table S6). We chose proteins that had 95%, 85%, 75%, and 65% homologies present percentage across 972 species, respectively, and then we re-generated the MMM protein pairs accordingly. The hit rates were calculated as *N*_*top*_∕*N*_*MMM*_, where *N*_*top*_ denoted the number of PPP predicted linkages under certain top rank threshold (ranging from 1 to 3,000) and *N*_*MMM*_ was the number of MMM re-generated protein pairs.

### Selection and vitalization of functional gene-sets and mitochondria correlation network

To evaluate the biological features of our whole proteome predicted functional linkages, we chose four different biological gene-sets databases: KEGG (Kanehisa et al., 2006), Biocarta, NCI/Nature Pathway Interaction Database (NCI) (Schaefer et al., 2009), and Reactome (Fabregat et al., 2016). The R/Bioconductor package “graphite” (Sales et al., 2012) re-constructed the pathway topology into 2,093 different protein-protein interaction networks. We calculated the predicted percentages for each gene-sets using our whole proteome functional linkages list. Three representative gene-sets as MAPK signalling pathway, GABA A receptor activation, and TCA citrate cycle, were visualized by an integrated Circos plot (Krzywinski et al., 2009).

To generate the human mitochondria correlation network with our prediction results, we retrieved 1,006 human mitochondria related proteins (Pagliarini et al., 2008), which was visualized by Cytoscape (Shannon et al., 2003). Five protein complexes in oxidative phosphorylation system (OXPHOS), including complex I (NADH-ubiquinone oxidoreductase), complex II (succinate-ubiquinone oxidoreductase), complex III (ubiquinol-cytochrome c reductase), complex IV (cytochrome c oxidase), and complex V (F1Fo ATP synthase) were highlighted, as well as MRPs.

### Statistical analysis

The pROC package was used to perform the typical ROC analysis (Robin et al., 2011). The SVD normalization was carried out by the SVD-Phy package (Franceschini et al., 2015). The rest programming tasks were conducted using the open-source R Project (http://www.r-project.org/).

##  Supplemental Information

10.7717/peerj.3712/supp-1 Supplemental Information 1 Supplemental Tables 1–2

10.7717/peerj.3712/supp-2 Supplemental Information 2 Supplemental Table 3

10.7717/peerj.3712/supp-3 Supplemental Information 3 Supplemental Tables 4–7

10.7717/peerj.3712/supp-4 Figure S1 Validation of PPP performance ROC curves and PR curves are generated by using another random negative reference dataset (A–B), complexes with less than 40 subunits (C–D), and a validation dataset from Ta and colleagues (Ta, Koskinen & Holm, 2011) (E–F).

10.7717/peerj.3712/supp-5 Figure S2 Hit rates of PPP predicted linkages among the MMM protein pairs The protein pairs with more homology across a wide range of species showed a higher hit rate at more stringent top ranks, indicating increased true linkage detection by PPP. The hit rate denotes the ratio between the number of linkages predicted by PPP and the number of MMM linkages.

10.7717/peerj.3712/supp-6 Figure S3 The phylogenetic plot of selected mitochondrial proteins The two clusters consist of a group of proteins present among a wide range of species and a group that was almost exclusively present in Metazoa.

## Additional Information and Declarations

### Data Availability

The following information was supplied regarding data availability:
http://prephylopro.org/phyloprofile/.
