# Supplementary figures and images for: PrePhyloPro: phylogenetic profile-based prediction of whole proteome linkages
Source: PeerJ. 2017 Aug 28;5:e3712. doi: 10.7717/peerj.3712 (PMC5578374; doi:10.7717/peerj.3712)

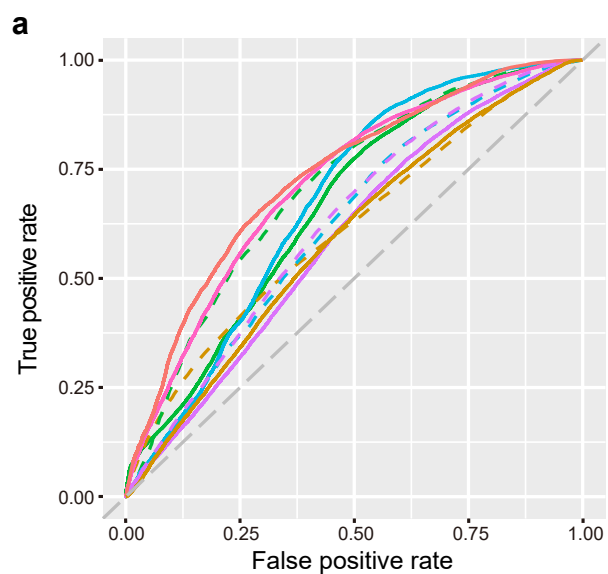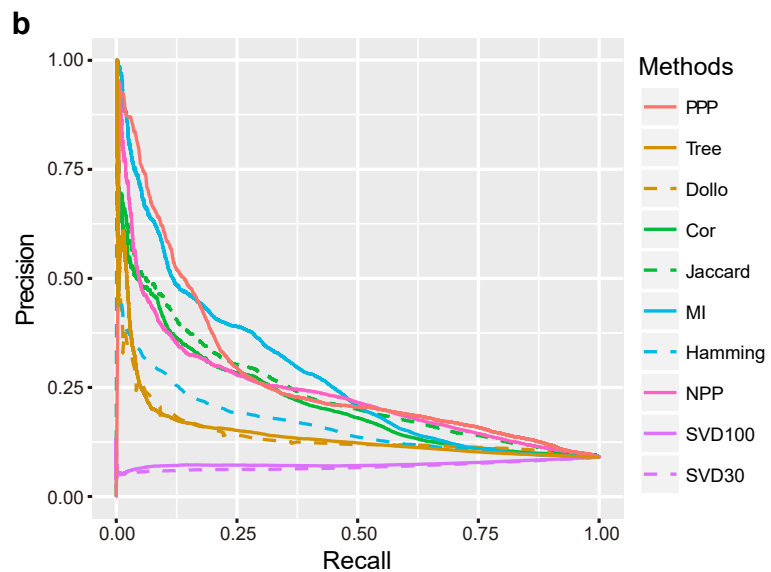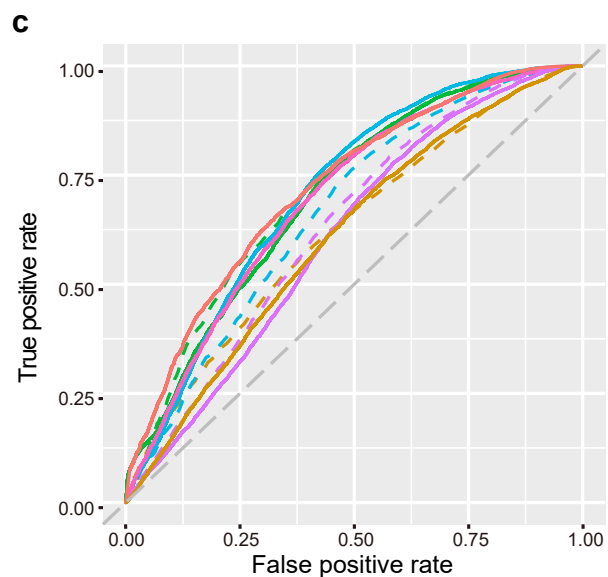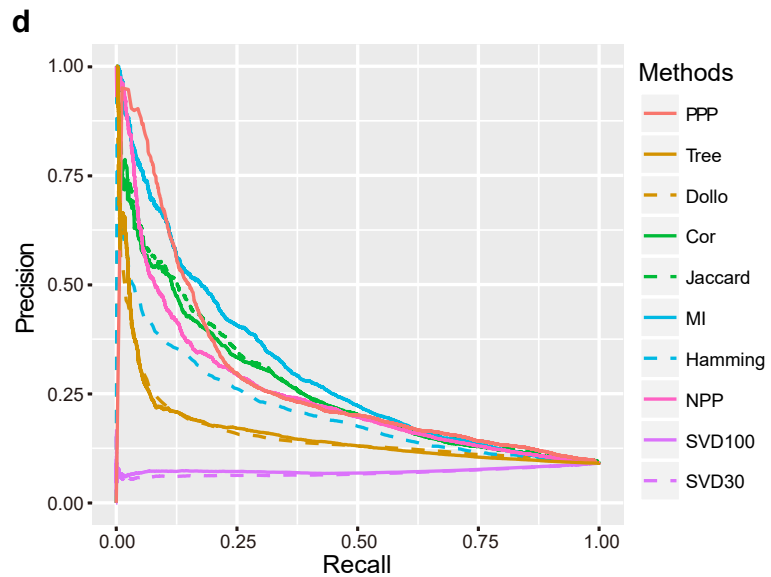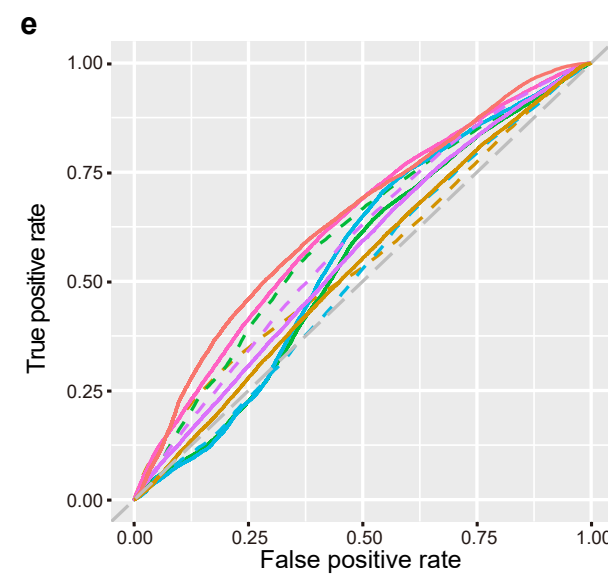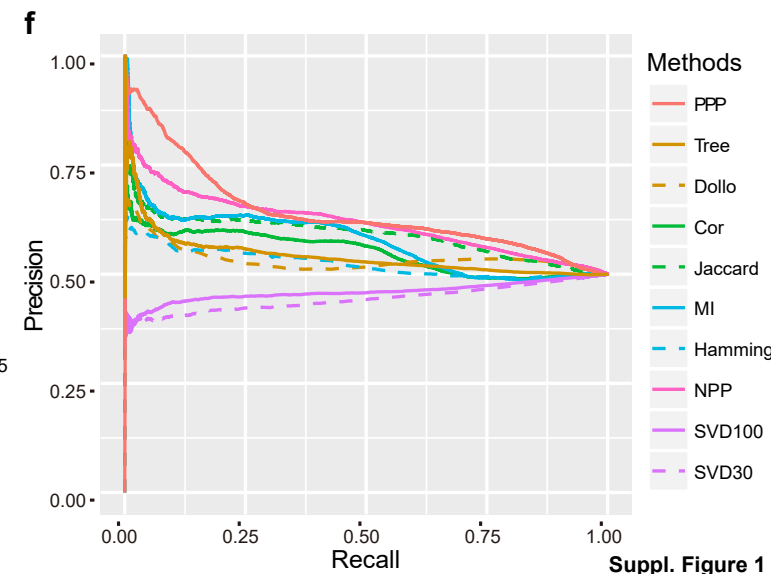

Suppl. Figure 1

Supplement: Figure S1 — ROC curves and PR curves are generated by using another random negative reference dataset (A–B), complexes with less than 40 subunits (C–D), and a validation dataset from Ta and colleagues (Ta, Koskinen & Holm, 2011) (E–F). [file peerj-05-3712-s004.pdf]

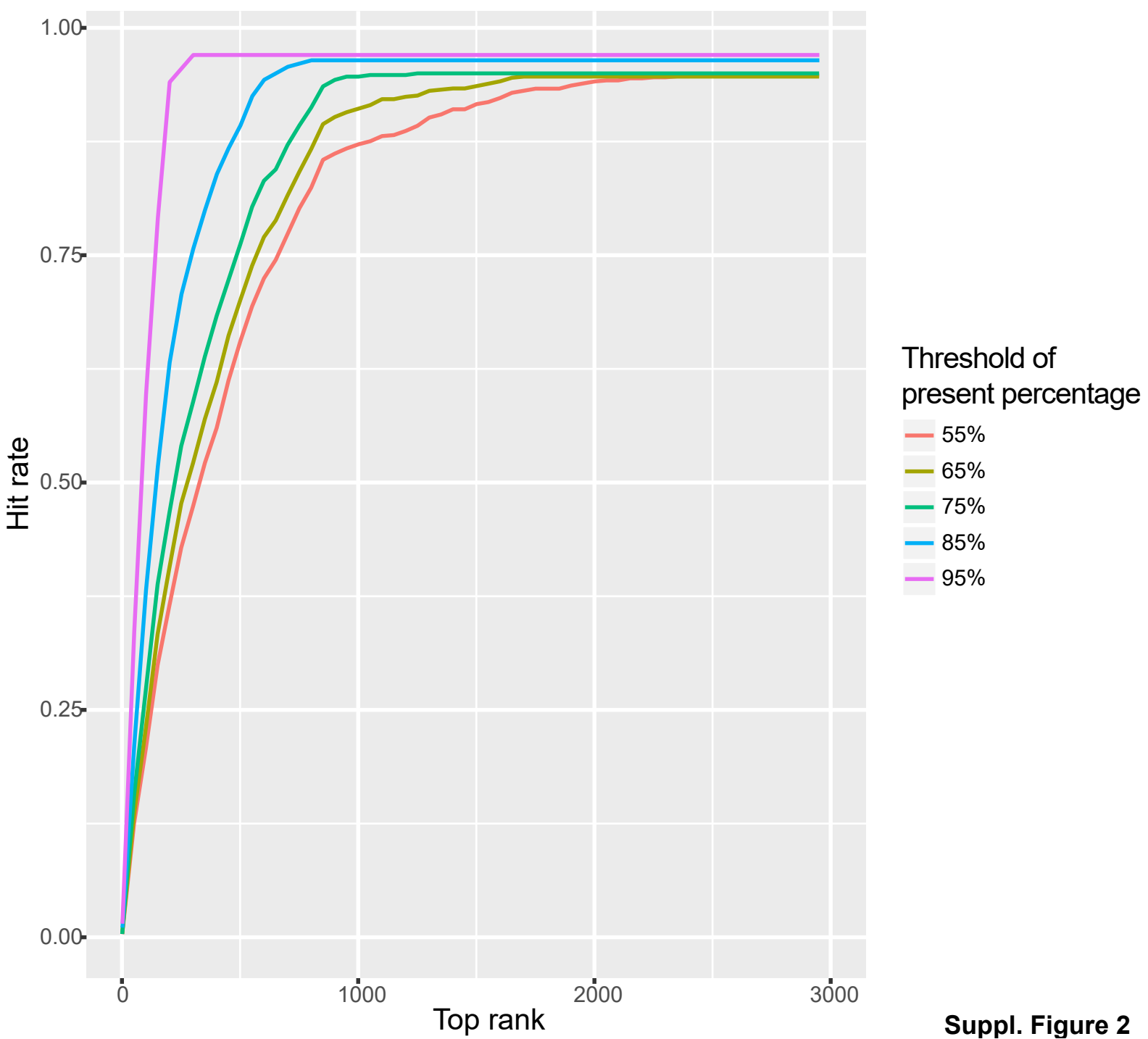

Supplement: Figure S2 — The protein pairs with more homology across a wide range of species showed a higher hit rate at more stringent top ranks, indicating increased true linkage detection by PPP. The hit rate denotes the ratio between the number of linkages predicted by PPP and the number of MMM linkages. [file peerj-05-3712-s005.pdf]

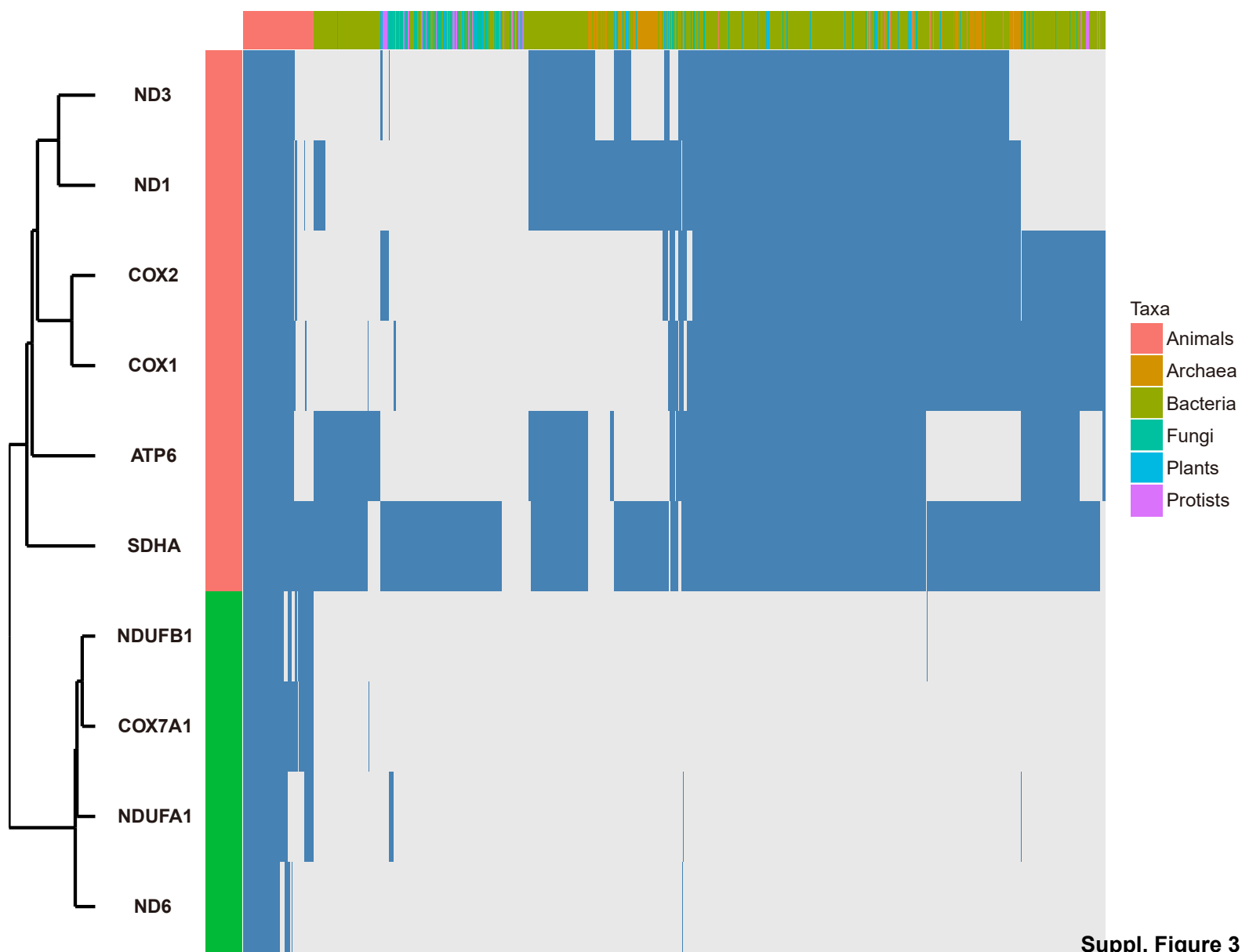

Suppl. Figure 3

Supplement: Figure S3 — The two clusters consist of a group of proteins present among a wide range of species and a group that was almost exclusively present in Metazoa. [file peerj-05-3712-s006.pdf]
